# Circulating Extracellular Vesicle-Derived microRNAs as Novel Diagnostic and Prognostic Biomarkers for Non-Viral-Related Hepatocellular Carcinoma

**DOI:** 10.3390/ijms242216043

**Published:** 2023-11-07

**Authors:** Bootsakorn Boonkaew, Nantawat Satthawiwat, Nutcha Pinjaroen, Natthaya Chuaypen, Pisit Tangkijvanich

**Affiliations:** 1 Center of Excellence in Hepatitis and Liver Cancer, Department of Biochemistry, Faculty of Medicine, Chulalongkorn University, Bangkok 10330, Thailand; bootsakorn.b@gmail.com (B.B.); 6371007030@student.chula.ac.th (N.S.); 2 Department of Radiology, Faculty of Medicine, Chulalongkorn University, Bangkok 10330, Thailand; nutcha.p@chula.ac.th

**Keywords:** extracellular vesicles, nonalcoholic fatty liver disease, hepatocellular carcinoma, microRNAs, biomarker

## Abstract

Extracellular vesicle-derived microRNAs (EV-miRNAs) are promising circulating biomarkers for chronic liver disease. In this study, we explored the potential significance of plasma EV-miRNAs in non-hepatitis B-, non-hepatitis C-related HCC (NBNC-HCC). We compared, using the NanoString method, plasma EV-miRNA profiles between NBNC-HCC and control groups including patients with non-alcoholic fatty liver disease (NAFLD) and healthy controls. The differentially expressed EV-miRNAs were validated in another set of plasma samples by qRT-PCR. A total of 66 significantly differentially expressed EV-miRNAs between the HCC and the control groups were identified in the discovery set. In the validation cohort, including plasma samples of 70 NBNC-HCC patients, 70 NAFLD patients, and 35 healthy controls, 5 plasma EV-miRNAs were significantly elevated in HCC, which included miR-19-3p, miR-16-5p, miR-223-3p, miR-30d-5p, and miR-451a. These miRNAs were found to participate in several cancer-related signaling pathways based on bioinformatic analysis. Among them, EV-miR-19-3p exhibited the best diagnostic performance and displayed a high sensitivity for detecting alpha-fetoprotein-negative HCC and early-stage HCC. In multivariate analysis, a high EV-miR-19-3p level was demonstrated as an independently unfavorable predictor of overall survival in patients with NBNC-HCC. In conclusion, our data have indicated, for the first time, that EV-miR-19-3p could serve as a novel circulating biomarker for the diagnosis and prognosis of NBNC-HCC.

## 1. Introduction

Hepatocellular carcinoma (HCC) is a heterogenous tumor with the majority of cases occurring in the setting of underlying chronic liver disease (CLD) [1]. Although chronic viral hepatitis has been the major risk factor for HCC, an increasing proportion of patient disease is attributable to non-hepatitis B-, non-hepatitis C-related HCC (NBNC-HCC) as a result of the growing burden of non-alcoholic fatty liver disease (NAFLD) [1]. Currently, NAFLD is considered to be a leading cause of HCC in most Western countries. In Asian populations, the prevalence of NAFLD is also becoming an important public health concern that potentially leads to progressive liver disease including cirrhosis and HCC [2]. Early detection of HCC enhances the likelihood of curative treatment by surgical resection, liver transplantation, or local ablation. However, the overall prognosis of HCC remains unsatisfactory because of the biologic aggressiveness of the tumor and high rates of recurrence after therapies [3]. Moreover, NBNC-HCC tends to be detected at a late tumor stage, which could lead to worse prognosis in comparison to virus-related HCC [4]. A recent multicenter study demonstrated that there was no survival improvement among patients with NBNC-HCC over the past two decades while the survival rate of viral-related HCC considerably increased [5]. Therefore, reliable circulating biomarkers for early diagnosis and prognostic prediction are urgently required to improve the clinical outcomes of patients with NBNC-HCC.

In recent years, ‘liquid biopsy’ has emerged as a novel method for the characterization of circulating cancer components, and provides a strong basis for precision oncology in terms of early diagnosis, therapeutic monitoring, and prognostication [6]. Among various liquid-biopsy based techniques, extracellular vesicles (EVs) are promising circulating biomarkers for HCC [6]. EVs are membrane-bound organelles produced by cells that are classified based on size and biogenesis process. Exosomes (50–150 nm) originate from multivesicular bodies, microvesicles (100–1000 nm) produce directly from membrane bubbling, and apoptotic bodies (500–2000 nm) generate from apoptotic process [7]. EVs serve as cargoes to mediate intercellular communication and are involved in various biological functions and disease progression by actively carrying proteins, lipids, and nucleic acids. Among them, microRNAs (miRNAs), small non-coding RNAs of 20–22 nucleotides, have attracted more attention because of their regulatory roles in a number of key pathophysiological processes [8]. Additionally, circulating enriched EV-derived miRNAs (EV-miRNAs) appear to be more stable and homogeneous than free miRNAs in serum/plasma [9]. Thus, EV-miRNAs, as opposed to free miRNAs, are considered to be more specific and better candidates as cancer biomarkers [10]. Although several ‘free-circulating’ miRNAs have been shown to be useful in distinguishing HCC from non-HCC, available data regarding the role of EV-miRNAs are limited, particularly in NBNC-HCC.

In the present study, we aimed to explore the potential clinical significance of EV-miRNAs in patients with NBNC-HCC. First, we compared the profiles of plasma EV-miRNAs of the NBNC-HCC and non-cancerous groups, including patients with NAFLD and healthy controls using the NanoString technique. Additionally, the differentially expressed EV-miRNAs were validated in another set of plasma samples by quantitative Real-Time PCR (qRT-PCR) to identify novel biomarkers for NBNC-HCC. Finally, the diagnostic and prognostic roles of these potential biomarkers were analyzed.

## 2. Results

### 2.1. Characteristics of the Participants

To construct miRNA profiling in the discovery set, 9 plasma samples per group of patients with NBNC-HCC, NAFLD, versus heathy controls were analyzed by the NanoString miRNA assay. Moreover, the quantitative levels of candidate miRNAs were validated by qRT-PCR in the plasma samples of 35 healthy controls, 70 patients with NAFLD, and 70 patients with NBNC-HCC. Baseline characteristics of the participants in the validated cohort are shown in Table 1.

### 2.2. Characterization of EVs

**Figure 1:**
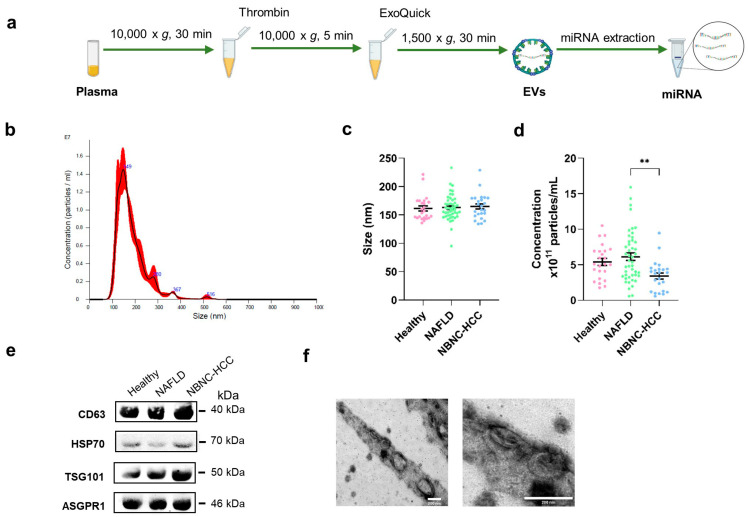
Schematic diagram of the study protocol and characterization of EVs derived from plasma. (**a**) Diagram of plasma EV isolation protocol, (**b**) Representative nanoparticle tracking plot for size distribution from a sample, (**c**) Quantification of particle size diameter (nm), and (**d**) Concentration (particles/mL) of plasma EVs from the healthy control, NAFLD, and NBNC-HCC groups. (**e**) Expression of EV markers, CD63, HSP70, and TSG101, and hepatocyte-specific receptor, ASGPR1 by Western blotting. (**f**) Transmission electron microscopy images of EVs from a sample. Scale bars, 200 μm. Data are presented as means ± S.E.M, ** *p* < 0.01.

To characterize EVs isolated from plasma samples (Figure 1a), the size and concentration of EVs were first determined by nanoparticle tracking analysis (NTA). As expected, the average size of particles in overall samples was 162.9 ± 22.1 nm in diameter (Figure 1b,c). The concentrations of isolated EVs from healthy controls were similar to the NAFLD group (5.40 × 10^11^ ± 2.39 × 10^11^ and 6.12 × 10^11^ ± 3.59 × 10^11^ particles/mL, respectively; Figure 1d), whereas the lowest concentration of EVs (3.41 × 10^11^ ± 2.15 × 10^11^ particles/mL) was found in samples from patients with NBNC-HCC. To verify the EV markers, Western blot analysis was then performed. Our results showed that the EV-enriched proteins, including CD63, heat shock protein 70 (HSP70), and the tumor-susceptibility gene 101 (TSG101), were expressed in isolated EV samples. A hepatocyte-specific receptor or asialoglycoprotein receptor 1 (ASGPR1) was also identified to verify that the isolated EVs were partially hepatocyte-derived EVs (Figure 1e). In addition, the particle diameter and the morphology were confirmed and visualized by transmission electron microscopy (TEM). These vesicles were less than 200 nm in size, with a lipid bilayer, indicating that they were EVs as described previously [11] (Figure 1f). We next evaluated whether the isolated EVs provided intra-vesicular miRNAs. Our results were in line with previous data demonstrating that there was no statistically significant difference between *C*_t_ values of miRNAs (such as miR-26a-5p, miR-223-3p, and let-7a-5p) in EVs treated with RNase and without RNase-A (Figure S1a–c) [12,13]. Together, these results indicated that our protocol could specifically identify EV-miRNAs, in accordance with previous data [11]; thus, we did not perform RNase-A treatment for the subsequent experiments.

### 2.3. Profiling of EV-Derived microRNAs

To explore the profiles of EV-miRNAs, we performed the expression of 800 miRNAs using NanoString platform (nCounter Human v3 miRNA expression assay) on the discovery set of patients with NBNC-HCC, NAFLD, and healthy controls. Raw data and normalized data outputs from nSolver 4.0 Software are available in Supplementary S1 File.

**Figure 2:**
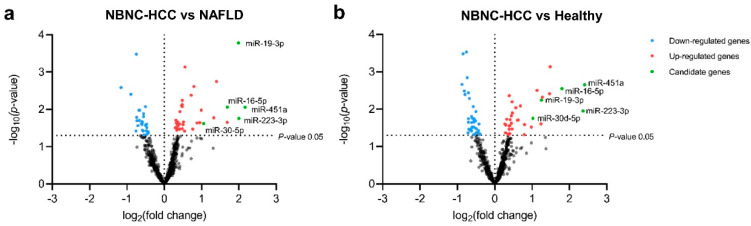
Transcriptome profiling of miRNAs from plasma EVs using NanoString microarray. (**a**) Volcano plot of all differentially expressed miRNAs in the NBNC-HCC samples compared with the NAFLD samples, and (**b**) the NBNC-HCC samples compared with healthy controls. The significantly up-regulated and down-regulated miRNAs are denoted as red and blue dots, respectively.

Based on differentially expressed miRNAs between groups (log_2_-fold change (FC) and *p* < 0.05), we found 66 significant differentially expressed miRNAs (DEmiRNAs) in patients with NBNC-HCC vs. NAFLD, which included 39 up- and 27 down-regulated miRNAs (Figure 2a), and 73 DEmiRNAs between patients with NBNC-HCC vs. healthy controls, including 36 up- and 36 down-regulated miRNAs (Figure 2b). Among the significantly up-regulated miRNAs, a Venn diagram showed that five miRNAs, including miR-19-3p, miR-16-5p, miR-223-3p, miR-30d-5p, and miR-451a, overlapped between NBNC-HCC vs. NAFLD and NBNC-HCC vs. healthy controls (Figure S2). Accordingly, these five up-regulated miRNAs were subsequently selected for further validation. The data for DEmiRNAs between patients with NAFLD and healthy controls are available in Figure S3.

### 2.4. Functional Gene Annotation and Pathway Enrichment Analysis

**Figure 3:**
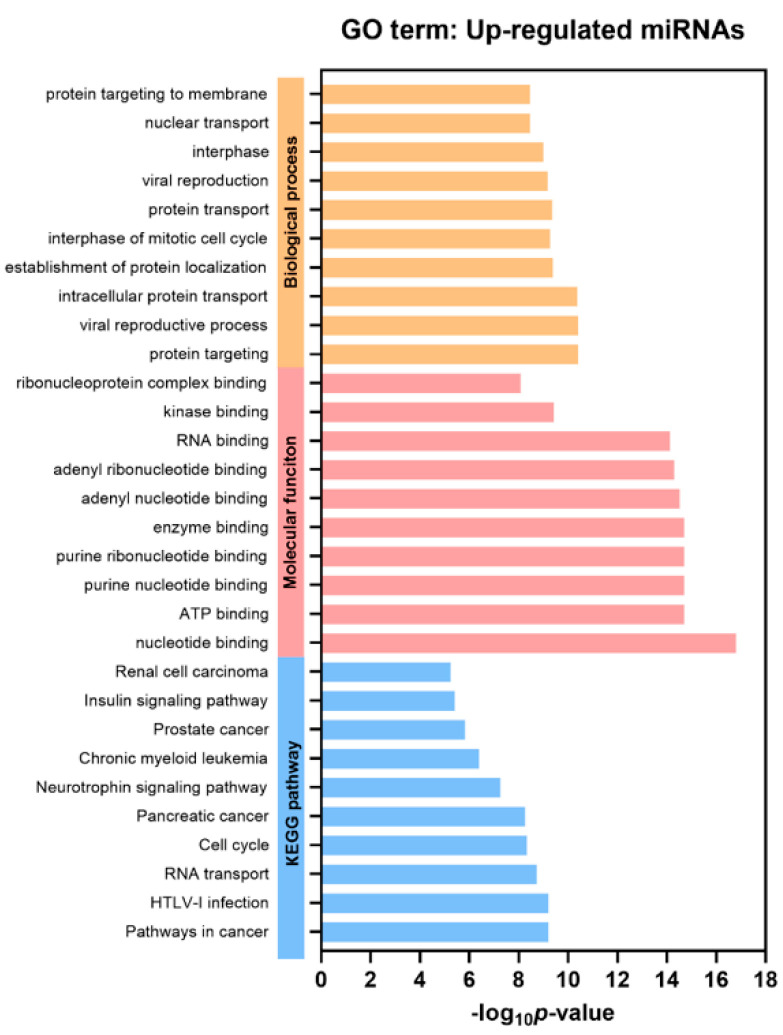
Gene Ontology (GO) analysis of the differentially up-regulated EV miRNAs. Top 10 significantly enriched GO terms of biological process, molecular function, and KEGG pathways (*p* < 0.05).

Gene Ontology (GO) and gProfiler analysis were performed to reveal enrichment of potential target genes of the significantly expressed miRNAs. The top 10 GO categories for the differentially up-regulated miRNAs and down-regulated miRNAs are demonstrated in Figure 3 and Figure S4 (*p* < 0.05), respectively. These significantly up-regulated and down-regulated miRNAs were involved in several biological processes, such as protein targeting and transport, nuclear transport, and cell cycle. Regarding molecular function analysis, these miRNAs were found to be enriched in nucleotide, ATP, and enzyme binding. Furthermore, Kyoto Encyclopedia of Genes and Genomes (KEGG) pathways indicated that the significantly up-regulated miRNAs participated in pathways in cancer, similar to those with down-regulated miRNAs.

### 2.5. Plasma EV-miRNA Expression in the Validation Set

**Figure 4:**
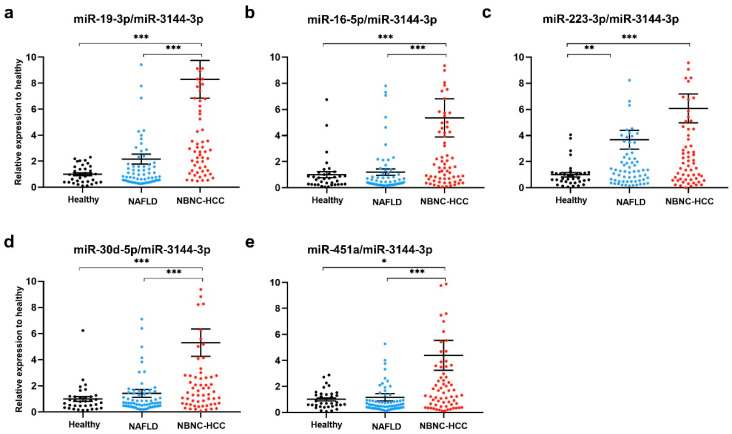
Validation of candidate miRNAs in plasma EV using qRT-PCR. The relative expressions of the following: (**a**) miR-19-3p, (**b**) miR-16-5p, (**c**) miR-223-3p, (**d**) miR-30d-5p, and (**e**) miR-451a, in plasma EVs of healthy controls (n = 35), patients with NAFLD (n = 70), and patients with NBNC-HCC (n = 70). Data are presented as mean ± S.E.M., normalized with a reference miRNA, miR-3144-3p, and expressed relative to those of healthy controls. * *p* < 0.05, ** *p* < 0.01 and *** *p* < 0.001.

To validate the above-mentioned five up-regulated candidate miRNAs, plasma EV- miRNAs from a total of 175 participants, including 35 healthy controls, 70 patients with NAFLD, and 70 patients with NBNC-HCC were evaluated by qRT-PCR using miR-3144-3p normalization. The results showed that all miR-19-3p, miR-16-5p, miR-223-3p, miR-30d-5p, and miR-451a expression levels were significantly higher in patients with NBNC-HCC compared with healthy controls (Figure 4a–e). When compared with the NAFLD group, the expression levels of miR-19-3p, miR-16-5p, miR-30d-5p, and miR-451a were significantly increased in the NBNC-HCC group (Figure 4a–e). Moreover, similar results of miRNA expression levels were found upon normalization with U6 (Figure S5a–e), suggesting consistent results of validated miRNAs across various internal controls. Overall, these findings indicate that plasma EV-miRNAs could effectively distinguish the NBNC-HCC from non-HCC groups.

Besides plasma EVs, we also investigated the expression levels of candidate miRNAs in both tumor and adjacent non-tumor liver tissue samples obtained from patients with NBNC-HCC (n = 11 pairs) using qRT-PCR. Among candidate miRNAs, only miR-19-3p differed significantly between the tumor and adjacent non-tumor samples, 18.76 ± 33.31 vs. 1.00 ± 0.94 (*p* = 0.042) (Figure S6a). There was no significant difference found between the levels of miR-16-5p (0.63 ± 0.54 vs. 1.00 ± 0.91, *p* = 0.700), miR-223-3p (1.05 ± 1.31 vs. 1.00 ± 0.79, *p* = 0.465), miR-30d-5p (0.62 ± 1.26 vs. 1.00 ± 1.49, *p* = 0.175), and miR-451a (0.31 ± 0.46. vs. 1.00 ± 1.35, *p* = 0.148) (Figure S6b–e).

### 2.6. Diagnostic Role of Plasma EV-miRNAs

**Figure 5:**
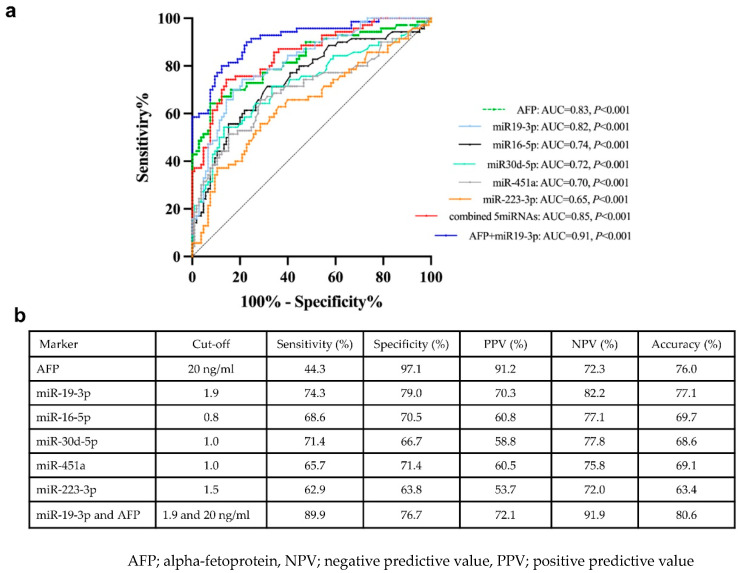
Receiver operating characteristic (ROC) curves of the candidate miRNAs for distinguishing between NBNC-HCC and non-HCC. (**a**) The area under the curve (AUC), and (**b**) the cut-off value and the discriminatory performance of each EV-miRNAs.

To evaluate the diagnostic performance of biomarkers in distinguishing between the NBNC-HCC and non-HCC groups, receiver operating characteristic (ROC) curves were analyzed (Figure 5a). The area under the curve (AUC) was 0.82 (95% confidence interval (CI); 0.75–0.88, *p* < 0.001) for miR-19-3p, 0.74 (95% CI; 0.67–0.82, *p* < 0.001) for miR-16-5p, 0.65 (95% CI; 0.56–0.73, *p* = 0.001) for miR-223-3p, 0.72 (95% CI; 0.64–0.80, *p* < 0.001) for miR-30d-5p, 0.70 (95% CI; 0.61–0.78, *p* < 0.001) for miR451a, and 0.83 (95% CI; 0.76–0.89, *p* < 0.001) for alpha-fetoprotein (AFP). In addition, the ROC curve for a combination of all EV-miRNAs was also examined. Our results showed that multiple miRNAs did not provide a better AUC than miR-19-3p alone. However, combined miR-19-3p and AFP increased performance for the diagnosis of HCC compared with miR-19-3p alone. The cut-off value and diagnostic performance of each EV-miRNAs, AFP, and the combination of miR-19-3p and AFP is shown in Figure 5b.

If categorized based on the normal upper limit of AFP (20 ng/mL), there were 39 (55.7%) and 31(44.3%) HCC patients showing AFP-negative and AFP-positive, respectively. In the AFP-negative group, 76.9% (30/39) of HCC patients had elevated circulating miR-19-3p levels (≥1.9), while in the AFP-positive group, high expression of miR-19-3p was found in 71.0% (22/31) of patients. Among early HCC cases (BCLC stage 0 and A), we found that 36.0% (9/25) of patients had elevated AFP levels, while 80.0% (20/25) of patients had high miR-19-3p expression. Together, these results might indicate that circulating EV-miR-19-3p was a promising biomarker for detecting AFP-negative HCC and early HCC, as well as a complementary to AFP in diagnosis of NBNC-HCC in our cohort.

### 2.7. Prognostic Role of Plasma EV-miRNAs Regarding Overall Survival

**Figure 6:**
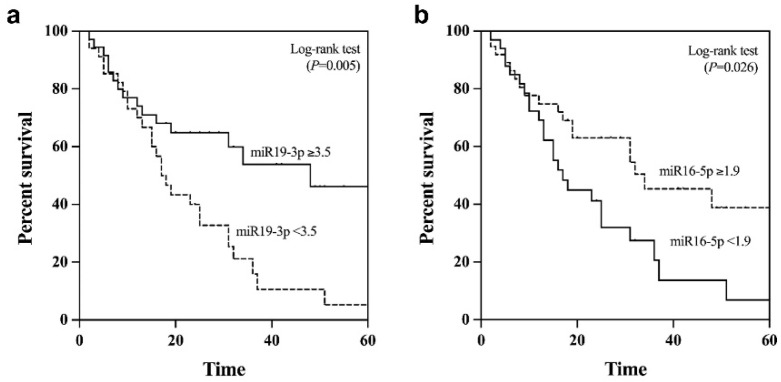
Kaplan–Meier survival curves for overall survival analysis of patients with NBNC-HCC (**a**) plasma EV-miR-19-3p, and (**b**) plasma EV-miR-16-5p.

Apart from its diagnostic value, we further examined the potential prognostic role of plasma EV-miR-19-3p in patients with NBNC-HCC. Using the median value as the cut-off level (3.5), the median overall survival of patients with miR-19-3p < 3.5 and ≥3.5 were 38.2 and 22.3 months, respectively (*p* = 0.05 by log rank test) (Figure 6a). For plasma EV-miR-16-5p, the median overall survival of HCC patients with low (<1.9) levels was significantly better than that of patients whose levels were elevated (35.7 vs. 23.1 months, *p* = 0.026) (Figure 6b). However, there was no significant difference in overall survival associated with the circulating levels of other EV-miRNAs, including miR-223-3p, miR-30d-5p, and miR-451a.

All 5 plasma EV-miRNAs were entered into the multivariate analysis together with other parameters that could influence overall survival of patients with NBNC-HCC. These variables included age, gender, serum TB, albumin, AST, ALT, platelet counts, AFP level, tumor size, and BCLC stage. The multivariate analysis based on the Cox regression analysis demonstrated that miR-19-3p, AFP, and BCLC stage were independent predictive factors for overall survival. However, miR-16-5p was not a parameter associated with overall survival (Table 2).

## 3. Discussion

The detection of HCC in early-stage cancer is an unmet clinical need; only 20–30% of patients are eligible for curative therapy mainly because of the lack of early-detection biomarkers. At present, serum AFP remains the most commonly used serum biomarker despite its insufficient performance in early detection of HCC. Overall, the sensitivity and specificity of AFP are approximately 60% and 80%, respectively, and its sensitivity decreases significantly in patients with early HCC [14]. Moreover, AFP levels remain normal (AFP level < 20 ng/mL) in up to 30% of advanced cancer cases but are elevated in some individuals without HCC, leading to high negative and false-positive rates. In this report, our data demonstrated that only 36% of early NBNC-HCC were AFP-positive (AFP level ≥ 20 ng/mL). Thus, additional novel biomarkers that could be used individually or in complement to AFP for better, more accurate detection of HCC are required.

In recent years, the potential role of EV-based liquid biopsy in the management of liver disease has been of great interest. Emerging evidence highlights the significance of EV-miRNAs in various chronic liver diseases, including viral hepatitis, NAFLD, alcohol-related liver disease, and HCC [15]. For instance, circulating EV-miRNA profiles could constitute non-invasive biomarkers for the assessment of severity in patients with NAFLD [16,17]. Regarding HCC, previous studies demonstrated that either single samples or panels of EV-miRNAs are potentially specific and sensitive biomarkers for the diagnosis of viral-related HCC [18,19]. However, data regarding the role of EV-miRNAs as novel biomarkers of NBNC-HCC are still needed. In this study, we initially characterized microtranscriptome to examine circulating EV-miRNA profiles in patients with NBNC-HCC by comparing with those of patients with NAFLD and healthy controls. In this discovery set, several differential expression profiles of EV-miRNAs between the HCC and control groups were revealed. In the validation set by qRT-PCR, plasma-derived EV-miRNAs, including miR-19-3p, miR-16-5p, miR-223-3p, miR-30d-5p, and miR-451a were significantly elevated in NBNC-HCC patients compared with the control group. Also, the data based on bioinformatics identified up- and down-regulated miRNAs associated with various biological processes, including protein targeting and transport, nuclear transport, and cell cycle. Moreover, the enriched KEGG pathways of DEGs were found to participate in several cancer-related signaling pathways.

In our report, we demonstrate for the first time that EV-miR-19-3p could be used as a promising biomarker for NBNC-HCC detection. We showed that EV-miR-19-3p had a high diagnostic ability in detecting AFP-negative cases. Additionally, the combined use of EV-miR-19-3p and AFP increased the diagnostic accuracy for NBNC-HCC. These findings suggest the potential use of EV-miR-19-3p as a sensitive biomarker for early HCC and a complementary biomarker for AFP-negative HCC. The clinical significance of the miR-19 family has been studied in various diseases. Of note, circulating EV-miR-19a-3p was recently identified as a novel biomarker among other miRNAs for early and non-invasive diagnosis of pancreatic cancer [20]. Moreover, a recent study showed that EV-miR-19a-3p was highly up-regulated in prostate cancer tissue specimens at the advanced stage, particularly after androgen stimulation [21]. Regarding its predictive role, Kaplan–Meier analysis also showed that high circulating EV-miR-19-3p was positively correlated with poor overall survival in patients with NBNC-HCC. Moreover, multivariate analysis verified that an increased EV-miR-19-3p level was an independently unfavorable predictor of overall survival. Collectively, our results provide evidence supporting a novel role of EV-miR-19-3p in the early detection and prognosis of NBNC-HCC patients.

Dysregulated expression of miR-19 has been shown to be involved in several types of solid tumors and represents one of the most investigated miRNAs in human cancer research [22]. Many studies have demonstrated that miR-19 plays a significant role in regulating and maintaining homeostasis of tissue function and immune regulation. Additionally, its dysregulation has been implicated in the pathogenesis and progression of tissue inflammation and fibrosis, as well as tumorigenesis [23]. For example, previous data suggests that serum miR-19a could be a biomarker for the early detection of colorectal cancer [24] and breast cancer [25]. Among studies related to HCC, most previous reports examine the expression of miR-19 in HCC cell lines or liver tissue specimens [26,27,28,29,30,31], with limited available data on blood-based samples [32]. For instance, miR-19 was shown to be up-regulated in tissue specimens and cell lines through the PTEN/Akt pathway in promoting HCC metastasis and chemoresistance [29,32]. In contrast, the expression level of miR-19a in human cancer specimens was significantly lower than that found in adjacent non-cancerous tissue, which might play an inhibitory role for HCC progression by targeting cyclin D1 [28]. This discrepancy might be due to several factors such as the etiologies and the heterogeneity of HCC, as well as differences in the studied HCC cell lines. Although miR-19 expression levels in HCC were rather inconsistent, a recent systematic review and meta-analysis revealed that up-regulated miR-19 expression was detected in HCC patients compared with non-malignant controls in most reports, indicating its crucial role in the diagnosis and prognosis of HCC [33]. Further studies are therefore required to elucidate the mechanism by which EV-miR-19-3p plays a crucial role in the development and progression of NBNC-HCC.

Our work had some limitations. The sample size was relatively small, and, thus, additional studies from multi-centers are required to confirm our findings. Secondly, although there are several potential etiologies for NBNC-HCC, the majority of our cases could likely be related to NAFLD, as other major causes of HCC including significant alcohol consumption were excluded at the initial enrollment. Moreover, most NBNC-HCC patients in our cohort had coexisting metabolic syndrome that was linked to NAFLD, as reported in most studies [34]. Thirdly, liver biopsy was not performed in patients with NAFLD. Although liver biopsy is currently the gold standard for diagnosis of NAFLD, this invasive method has limitations including sampling error, risk of complications, and is not feasible to perform in all patients. Instead, we used transient elastography, which is considered an accurate non-invasive tool to determine the severity of fibrosis and steatosis. Fourthly, this report was a cross-sectional cohort, and longitudinal studies to investigate the dynamic changes of the candidate EV-miRNAs should be further explored. Finally, one of the major challenges in applying EV miRNAs as diagnostic biomarkers is the lack of a standardized method for EV isolation [35]. In this study, we selected Exoquick^TM^ solution to enrich EVs from plasma. Compared with other techniques, this method is considered fast and easier to perform, and might be a better alternative for processing a large number of plasma samples in clinical setting. However, the potential drawback of this method is that it is non-selective and might yield low purity [15,36]. Despite these limitations, our results demonstrate that circulating EV-miR-19-3p is a reliable biomarker to differentiate between NBNC-HCC and non-HCC groups. Apart from its diagnostic role, plasma EV-miR-19-3p also serves as a good prognostic indicator for NBNC-HCC. This novel circulating biomarker might serve as a promising tool for the diagnosis and prognosis prediction of NBNC-HCC.

## 4. Materials and Methods

### 4.1. Research Subjects and Participant Consent

Blood samples for the assessment of EV-miRNAs were obtained from patients with NBNC-HCC who were followed-up at King Chulalongkorn Memorial Hospital (Bangkok, Thailand). All patients enrolled in this study were seronegative for HBsAg and anti-HCV, had no significant alcohol consumption (defined as >20 g ethanol/day in males and >10 g ethanol/day in females), and no coexisting causes of other chronic liver diseases such as autoimmune hepatitis, primary biliary cholangitis, or Wilson’s disease. HCC was diagnosed on the basis of typical findings on imaging studies and/or histopathology according to the American Association for the Study of Liver Diseases (AASLD) guideline [37]. Briefly, diagnostic criteria with dynamic imaging were established by findings of focal lesions with hyper-attenuation at the arterial phase and hypo-attenuation at the portal phase. Liver biopsy or fine needle aspiration was performed in case of uncertain diagnosis after imaging studies. Baseline clinical parameters were recorded, including tumor staging classified by the BCLC staging system [38]. Blood and tissue samples were collected from patients prior to any HCC therapy, including liver resection, radiofrequency ablation and transarterial chemoembolization. Moreover, the OS of patients with NBNC-HCC defined by the interval between initial assessment and death or the last follow-up visit was documented.

Patients with NAFLD, who had no evidence of HCC, as well as other liver disease, and who were seronegative for both HBsAg and anti-HCV, were included as a control group. The diagnosis of NAFLD was according to the AASLD criteria as determined by the controlled attenuation parameter (CAP) using a FibroScan device (Echosens, Paris, France), with the cut-off >248 dB/m [39,40]. Among this group of patients, current and past daily alcohol intake was less than 20 g/week and none of the patients received any steatogenic medication. Additionally, individuals who had no underlying disorders and had normal vibration-controlled transient elastography and CAP values served as healthy controls. The study was conducted according to the Declaration of Helsinki. The protocol was approved by the Institutional Review Board (IRB) of the Faculty of Medicine, Chulalongkorn University, and all participants signed informed consent forms before collection of the samples.

### 4.2. Blood Collection and Plasma Processing

Blood samples obtained from each subject were processed by centrifugation at 12,000× *g* for 30 min at 4 °C and then stored at −80 °C until analysis for miRNA profiles in the discovery cohort using NanoString^®®^ nCounter miRNA Expression Assay (NanoString Technologies, Seattle, WA, USA) and the validation of miRNAs using the qRT-PCR technique (Applied Biosystems, Waltham, MA, USA).

### 4.3. EV Isolation

EVs were extracted from plasma samples using the ExoQuick™ Exosome Isolation Kit (SBI, System Biosciences, Palo Alto, CA, USA) according to the manufacturer’s protocol. Briefly, for collecting the clear supernatant of plasma, 1 mL of the samples were incubated for 5 min with 8 µL of thrombin (final concentration of 5 U/mL) before centrifugation at 10,000× *g* for 5 min at 4 °C. Next, 250 µL of ExoQuick™ was added and incubated at 4 °C for 30 min. The mixture of ExoQuick™-plasma samples were centrifuged to precipitate EVs at 1500× *g* for 30 min. The pellet was then resuspended in 0.22 µm-filtered 1× PBS and stored at −80 °C until further use. For validation, 200 µL of plasma samples were used.

### 4.4. EV Characterization

#### 4.4.1. NTA

To characterize EVs from plasma, the quantity and size distribution of EVs in plasma samples were carried out using the NTA (Malvern Instruments, Malvern, UK). EVs were diluted 1000-fold for detecting between 50 and 100 particles per frame. Three 40-s videos were recorded with screen gain 3 and camera level 9 followed by an analysis of the data using NanoSight software (NTA 3.4 Build 3.4.003) with screen gain 9 and detection threshold 3.

#### 4.4.2. TEM

To characterize the morphology and size of EVs, a 5 µL drop of the suspension was loaded onto a 400 mesh formvar/carbon-coated grid (Electron Microscopy Sciences, Hatfield, PA, USA). To enhance the contrast between EVs and the background, grids were negatively stained with 2.5% uranyl acetate for 5 min. The excessive stain was blotted, and the grid was dried. Images were visualized under a transmission electron microscope using JEM-1400plus TEM (JEOL, Tokyo, Japan) at 80 kV.

#### 4.4.3. Western Blotting

To detect EV protein markers by immunoblotting, EV samples were lysed using RIPA buffer with Proteinase and Phosphatase Inhibitor Cocktail (Merck, Rahway, NJ, USA). The lysates were sonicated with 7 sets of 3-s pulses using Sonics Vibra-Cell™ (Sonics & Materials, Newtown, CT, USA). EV proteins (10 µg) were measured using Pierce™ BCA Protein Assay Kit (ThermoFisher Scientific, Waltham, MA, USA.) and loaded onto sodium dodecyl polyacrylamide gel electrophoresis. The proteins were then transferred to nitrocellulose membranes, blocked for 1 h with 5% BSA, and incubated at 4 °C overnight with primary antibodies against TSG101 (1:1000) (Ab83, 4A10) (Abcam, Boston, MA, USA), HSP70 (1:1000) (4876, D69, Cell signaling), CD63 (1:2000) (Ab193349, MX-49.129.5, Abcam). To identify hepatocyte-specific markers, ASGPR1 (1:1000) (SC-52623, 8D7, Santa Cruz) was used [41,42]. Following this process, the membrane was stained with horseradish peroxidase-conjugated secondary antibodies for 1 h. Antigen-antibody reactions were visualized with an enhanced chemiluminescence detection reagent and images were acquired using a ChemiDoc Imaging System (Bio-Rad Laboratories, Hercules, CA, USA).

### 4.5. NanoString miRNA Expression Analysis

To extract miRNA from EVs, isolated EVs from the above-mentioned method was extracted using miRNeasy Serum/Plasma Kit (Qiagen, Hilden, Germany). Before applying to NanoString analysis, miRNA was concentrated and contamination minimized using an Amicon Ultra YM-3 filter (Merck Millipore, Burlington, NJ, USA). Briefly, 320 µL of RNase-free water was added to the isolated miRNA, loaded onto the filter, and centrifuged at 14,000× *g* at 25 °C for 90 min. Three µL of the concentrated miRNAs were subjected to human NanoString nCounter miRNA expression assay (NanoString Technologies, Seattle, WA, USA) using, according to the manufacturer’s instructions, the nCounter Human miRNA Panel v3 that evaluated 800 miRNAs. In brief, miRNAs were hybridized to capture and report probes at 65 °C for 18 h, followed by purification and quantification on the nCounter Prep Station and Digital Analyzer. The resulting data were analyzed by nSolver 4.0 software to obtain the count of individual miRNA. The miRNA data were calculated by normalization to the top 100 miRNA counts in each sample.

### 4.6. EV and Tissue miRNA Extraction

To extract miRNA from EVs, isolated EVs from the above-mentioned method were extracted using the miRNeasy Serum/Plasma Kit (Qiagen, Hilden, Germany). For miRNA extraction from tissues, approximately 30 µg of the liver tissue was used and the miRNAs were extracted using the RNeasy Fibrous Tissue Kit (Qiagen, Hilden, Germany), according to the manufacturer’s recommendations. The quantity and quality of miRNA were measured using the DeNovix DS-11 Spectrophotometer (DeNovix, Wilmington, DE, USA).

### 4.7. Real-Time Quantitative Reverse Transcription PCR (qRT-PCR)

The miRNAs were reverse transcribed to complementary DNA (cDNA) by the SL-poly (A) sequence GTCGTATCCAGTGCAGGGTCCGAGGTATTCGCACTGGATACGACAAAAAAAAAAAAAAAAAAVN using RevertAid First Strand cDNA Synthesis Kit (ThermoFisher Scientific). qRT-PCRs were performed in duplicate using the QPCR Green Master Mix HRox 4x (Biotechrabbit, Hennigsdorf, Germany). The reactions were detected by a QuantStudio 5 Real-Time PCR System (Applied Biosystems, Waltham, MA, USA). Reaction with no cDNA template was run as a negative control on every plate for each assay. Thermal cycling parameters were started with activation step at 95 °C for 10 min, followed by 40 cycles of denaturation at 95 °C for 15 s, and extension at 72 °C for 20 s with optimal annealing temperatures of each gene for 15 s. Primer sequences are listed in Table S1.

Four internal control miRNAs including U6, miR-26a-5p, miR-3144-3p, and miR-302d-3p from NanoString data were used to normalize the miRNA expressions. The results indicated that in the validation set of samples (n = 10 for healthy controls, n = 10 for NAFLD, and n = 9 for NBNC-HCC), all four miRNAs showed a similar Ct value, 29.92 ± 1.10, 28.06 ± 1.21, 31.987 ± 1.03, and 29.46 ± 1.17 for U6, miR-26a-5p, miR-3144-3p, and miR-302d-3p, respectively (Figure S7a–d). However, miR-3144-3p and U6 exhibited the lowest % coefficient of variation of 3.23 and 3.69 compared to miR-26a-5p and miR-302d-3p (4.30 and 3.98, respectively). In this respect, it was suggested that the expression of miR-3144-3p and U6 were the most constant in our sample set. Thus, these miRNAs were suitable for normalization in the validated samples and data were calculated by the 2^−ΔΔCT^ method.

### 4.8. Statistical Analysis

Data were analyzed by SPSS statistics version 22 (SPSS Inc., Chicago, IL, USA) and graph visualizations were constructed using GraphPad Prism 8.0 (GraphPad Software, La Jolla, CA, USA). To compare between groups, Chi’s square or Fisher’s exact test were applied for categorical variables and Student’s *t*-test or one-way ANOVA were used for quantitative variables. The diagnostic performance was evaluated using ROC curve and the AUC with sensitivity and specificity analysis. The Kaplan–Meier analysis and log-rank test were calculated for the survival analysis. In addition, the Cox regression was applied for identifying independent factors associated with overall survival of patients with NBNC-HCC. A *p*-value < 0.05 was considered statistically significant.

## Figures and Tables

**Table 1:** Baseline characteristic of the validation cohort in this study.

Baseline Characteristics	Healthy Controls	Patients with NAFLD	Patients with NBNC-HCC	*p*
	(n = 35)	(n = 70)	(n = 70)	
Age (years)	53.2 ± 5.3	50.7 ± 9.5	68.8 ± 11.4	<0.001
Gender (Male)	4 (11.4)	30 (42.9)	54 (77.1)	<0.001
Body mass index (kg/m^2^)	22.8 ± 2.6	26.9 ± 4.1	24.3 ± 4.2	<0.001
Presence of metabolic syndrome		33 (47.1)	50 (71.4)	0.006
Total bilirubin (mg/dL)		0.7 ± 0.6	0.8 ± 0.5	0.845
Serum albumin (g/dL)		4.0 ± 0.7	3.6 ± 0.5	<0.001 *
Aspartate aminotransferase (IU/L)		24.5 ± 6.8	34.5 ± 17.9	<0.001 *
Alanine aminotransferase (IU/L)		42.9 ± 42.8	60.8 ± 79.0	0.134
Alkaline phosphatase (IU/L)		81.4 ± 45.2	146.7 ± 171.3	<0.001 *
Platelet count (109/L)		225.4 ± 92.3	216.0 ± 104.7	0.157
Alpha fetoprotein (ng/mL)		3.0 ± 2.9	3423.3 ± 12,469.8	0.025 *
Controlled attenuation parameter (dB/m)	196.5 ± 23.6	304.0 ± 42.9	-	<0.001 *
Transient Elastography (kPa)	3.8 ± 0.8	6.1 ± 1.7	-	<0.001 *
BCLC stage (0-A/B/C)		-	25(35.7)/27(38.6)/18(25.7)	-

Data shown as mean ± SD, BCLC; Barcelona Clinic Liver Cancer, * *p* < 0.05.

**Table 2:** Variables associated with overall survival in patients with HCC.

Variables	Category	Overall Survival			
		Univariate Analysis		Multivariate Analysis	
		OR (95%CI)	*p*	OR (95%CI)	*p*
Age (years)	<60 vs. ≥60	0.62 (0.28–1.41)	0.254		
Gender	Male vs. Female	0.67 (0.32–1.42)	0.295		
Total bilirubin (mg/dL)	<1.2 vs. ≥1.2	0.77 (0.49–1.27)	0.303		
Serum albumin (g/dL)	<3.5 vs. ≥3.5	0.97 (0.46–2.04)	0.931		
Aspartate aminotransferase (IU/L)	<55 vs. ≥55	1.64 (1.83–3.23)	0.153		
Alanine aminotransferase (IU/L)	<50 vs. ≥50	1.00 (0.42–2.39)	0.998		
Platelet count (10^9^/L)	≥100 vs. <100	1.05 (0.37–2.97)	0.927		
Alpha fetoprotein (ng/mL)	<100 vs. ≥100	2.09 (1.08–4.08)	0.029 *	2.04 (1.01–4.13)	0.048 *
Tumor size (cm.)	<5.0 vs. ≥5.0	1.63 (0.88–3.04)	0.123		
BCLC stage	0-A vs. B vs. C	2.15 (1.38–3.36)	0.001 *	2.07 (1.29–3.32)	0.002 *
EV-miR-19-3p	<3.5 vs. ≥3.5	2.39 (1.26–4.52)	0.008 *	2.71 (1.19–6.19)	0.018 *
EV-miR-16-5p	<1.9 vs. ≥1.9	1.98 (1.07–3.69)	0.030 *	0.97 (0.44–2.13)	0.944
EV-miR-30d-5p	<2.0 vs. ≥2.0	1.56 (0.83–2.93)	0.171		
EV-miR-451a	<1.7 vs. ≥1.7	1.74 (0.94–3.23)	0.078		
EV-miR-223-3p	<2.5 vs. ≥2.5	1.71 (0.92–3.19)	0.091		

Data were expressed as odds ratio (OR) and 95% confidence intervals (CI). BCLC; Barcelona Clinic Liver Cancer. * *p* < 0.05.

## Data Availability

This published article and the Supplementary Materials contain all of the established data or analyzed data throughout this study. Raw data for quantitative analysis may be provided by corresponding authors upon reasonable inquiry. Raw and normalized NanoString microarray data were submitted to the Gene Expression Omnibus (GEO) (accession number: GSE244605, https://www.ncbi.nlm.nih.gov/geo/query/acc.cgi?acc=GSE244605, accessed on 1 November 2023).

## Supplementary Materials

The following supporting information can be downloaded at: https://www.mdpi.com/article/10.3390/ijms242216043/s1.
