# Supplementary material for: Circulating Extracellular Vesicle-Derived microRNAs as Novel Diagnostic and Prognostic Biomarkers for Non-Viral-Related Hepatocellular Carcinoma
Source: Int J Mol Sci. 2023 Nov 7;24(22):16043. doi: 10.3390/ijms242216043 (PMC10671272; doi:10.3390/ijms242216043)
Supplement: Supplementary file 1 [file ijms-24-16043-s001.zip › ijms-2587889-supplementary.pdf]

Supplementary Information

Circulating extracellular vesicle-derived microRNAs as novel diagnostic and prognostic biomarkers for non-viral-related hepatocellular carcinoma

Bootsakorn Boonkaew[1], Nantawat Satthawiwat[1], Nutcha Pinjaroen[2], Natthaya Chuaypen[1]*, Pisit Tangkijvanich[1]*

[1]Center of Excellence in Hepatitis and Liver Cancer, Department of Biochemistry, Faculty of Medicine, Chulalongkorn University, Bangkok, Thailand

[2]Department of Radiology, Faculty of Medicine, Chulalongkorn University, Bangkok, Thailand

*Correspondence:

Pisit Tangkijvanich, M.D., Center of Excellence in Hepatitis and Liver Cancer, Faculty of medicine, Chulalongkorn University, Bangkok, 10330, Thailand.

Email: pisittkvn@yahoo.com; Tel: +66 2 256 4482

*Co-correspondence:

Natthaya Chuaypen, PhD. Center of Excellence in Hepatitis and Liver Cancer, Faculty of medicine, Chulalongkorn University, Bangkok, 10330, Thailand.

Email: natthaya.c@chula.ac.th; natthaya.ch56@gmail.com; Tel: + 66 2 256 4482

Figure S1

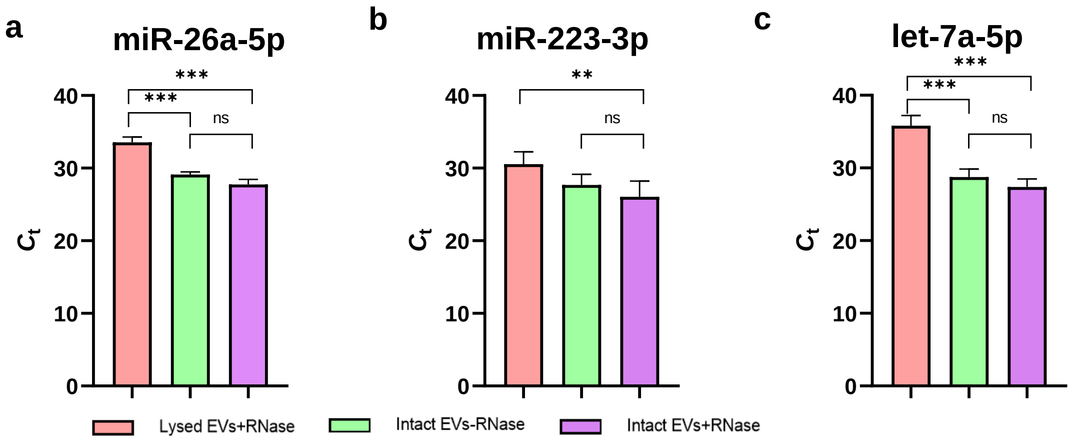

Figure S1. qRT-PCR analysis of EV miRNAs, (**a**) miR-26a-5p, (**b**) miR-223-3p, and (**c**) let-7a-5p upon RNase A treatment of lysed EVs and intact EVs with or without RNase A. Data are presented as means ± S.E.M of 5 independent samples; ns = not significant, $^{**}P < 0.01$, and $^{***}P < 0.001$.

Figure S2

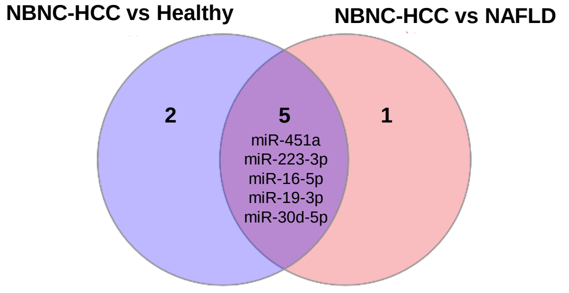

Figure S2. Venn diagram of intersect genes with fold change values more than 2.0 and showed a significant increase ($P < 0.05$) when pairwise comparison between NBNC-HCC and NAFLD, and NBNC-HCC and healthy controls.

Figure S3

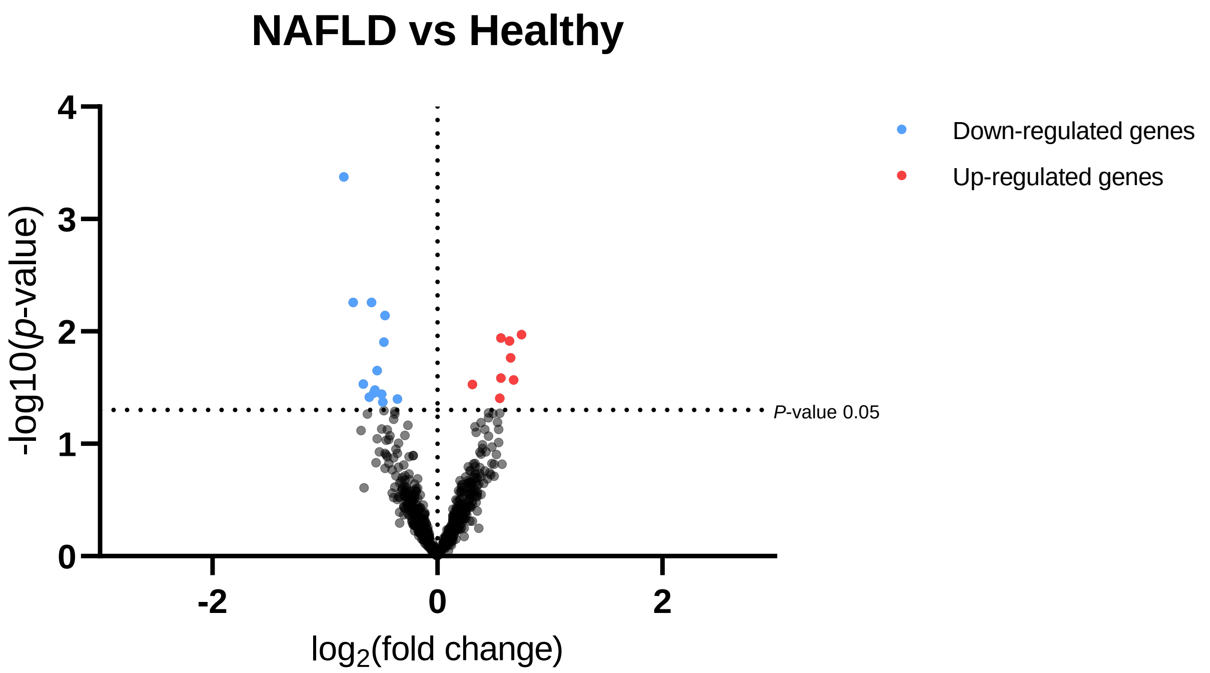

Figure S3. Volcano plot of all differentially expressed miRNAs in NAFLD samples compared with healthy control samples. The significantly up-regulated and down-regulated miRNAs are marked in red and blue dots, respectively.

Figure S4

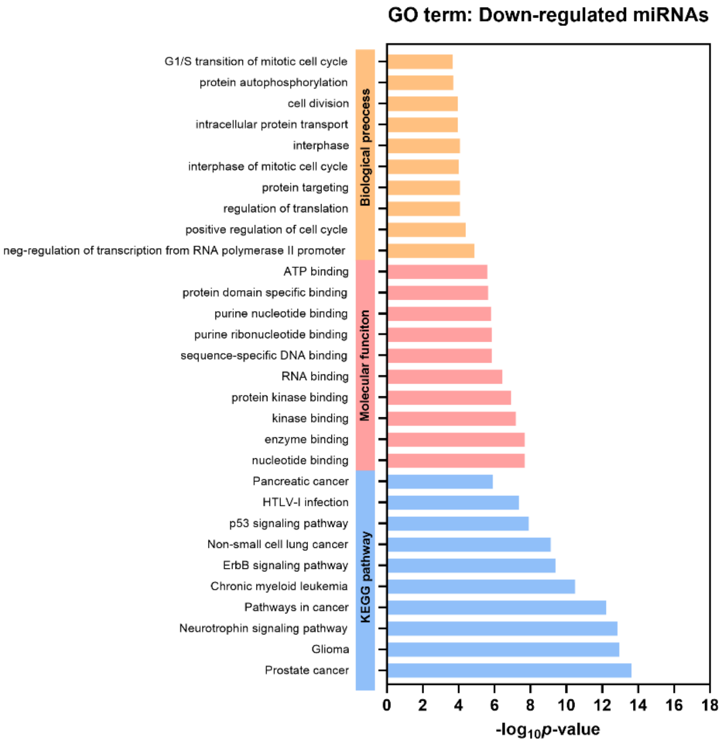

Figure S4. Gene Ontology (GO) analysis of the differentially downregulated EV miRNAs. Top 10 significantly enriched GO terms of biological process, molecular function, and KEGG pathways ($P < 0.05$).

Figure S5

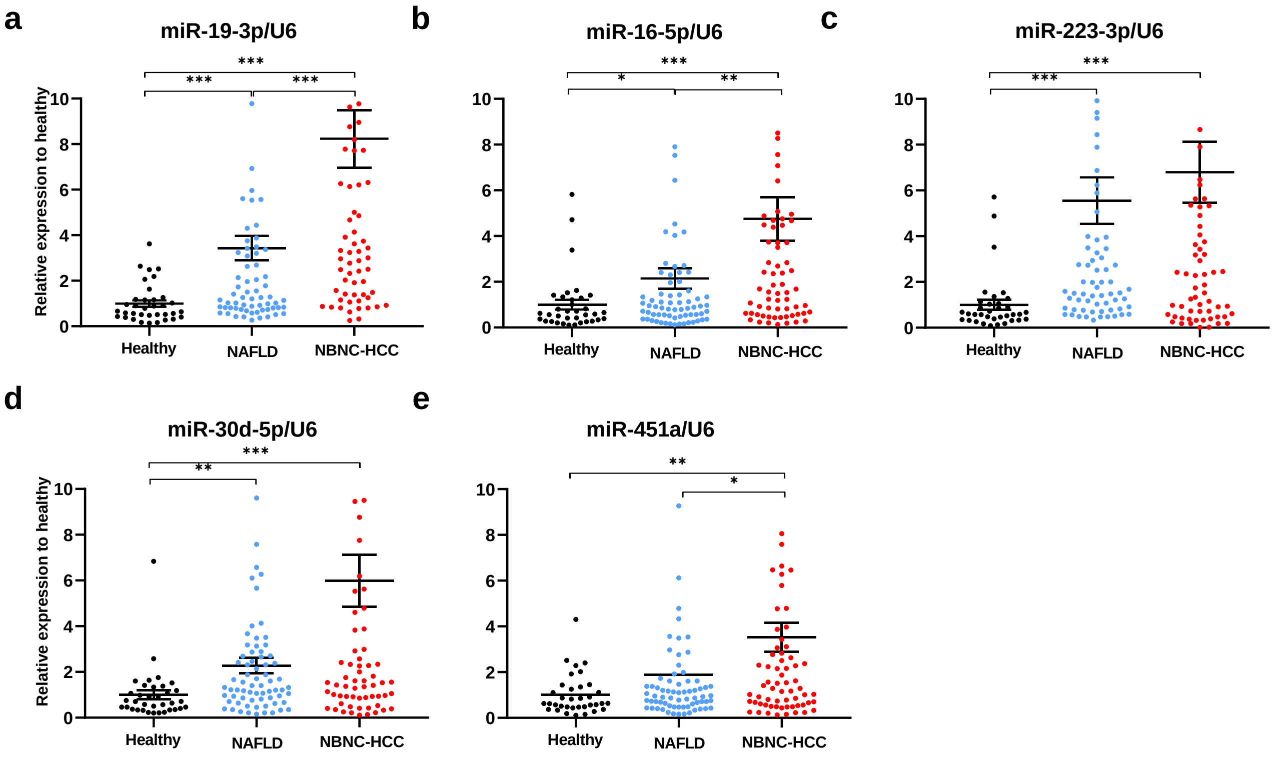

Figure S5. Validation of candidate miRNAs in plasma EV using qRT-PCR. The relative expressions of (**a**) miR-19-3p, (**b**) miR-16-5p, (**c**) miR-223-3p, (**d**) miR-30d-5p and (**e**) miR-451a in plasma EVs of healthy controls (n = 35), patients with NAFLD (n = 70), and patients with NBNC-HCC (n = 70). Data are presented as mean ± S.E.M., normalized with a reference gene, U6, and expressed relative to those of healthy controls. * $P < 0.05$, **$P < 0.01$ and *** $P < 0.001$.

Figure S6

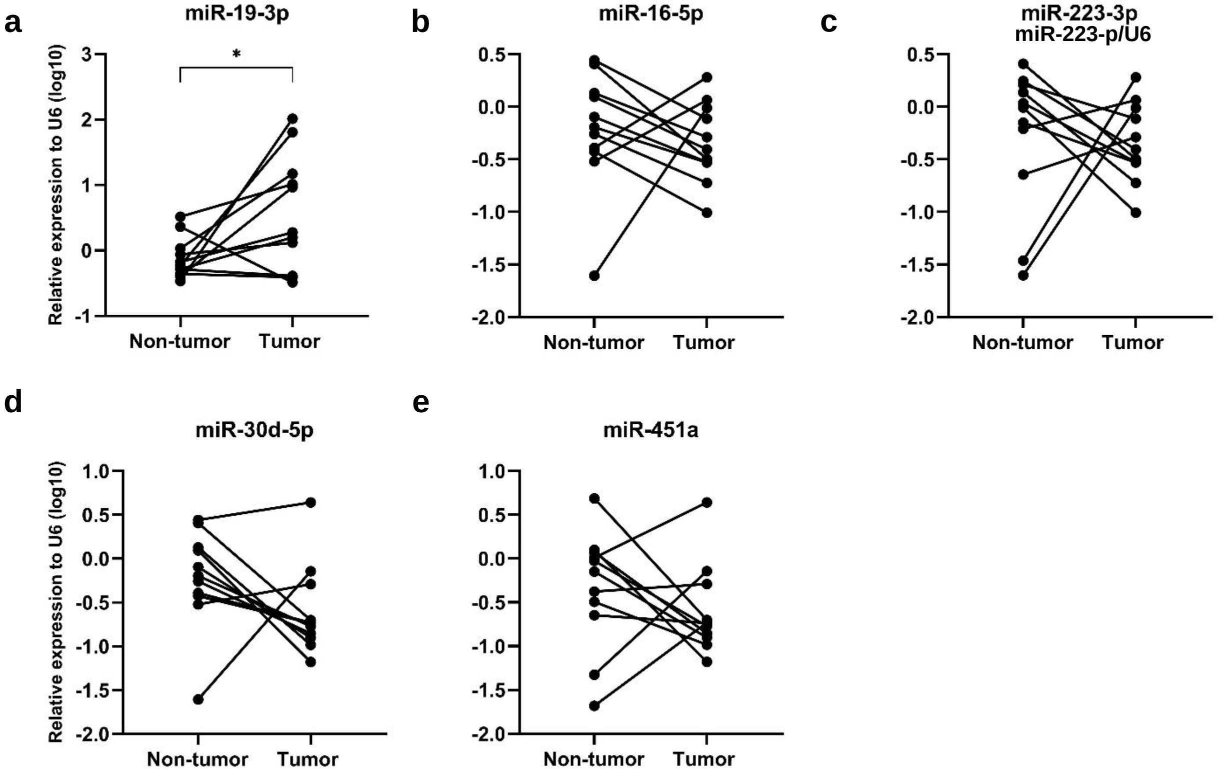

Figure S6. Expression of candidate miRNAs in tumor and adjacent non-tumor liver tissue samples using qRT-PCR. The expressions of (a) miR-19-3p, (b) miR-16-5p, (c) miR-223-3p, (d) miR-30d-5p, and (e) miR-451a in paired tissue samples (n = 11 pairs), normalized with a reference gene, U6. Data were analyzed using paired Student's t-test.

Figure S7

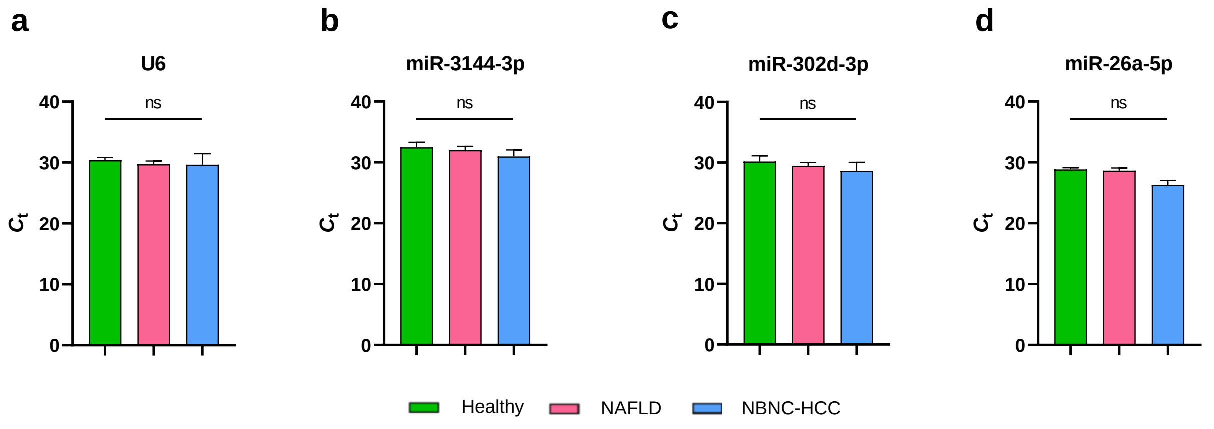

Figure S7. qRT-PCR analysis of candidate internal controls in the study cohort from plasma EVs of healthy controls (n = 10), NAFLD (n = 10), and NBNC-HCC (n = 9). Data are presented as means ± S.D.; ns = not significant.

Table S1. Sequences of primers used for qRT-PCR analysis

Sequence	Sequence 5’-3’	Tm (°C)
miR-451a	AAACCGTTACCATTACTGAGTT	52
miR-223-3p	TGTCAGTTTGTCAAATACCCCA	55
miR-19-3p	TGTGCAAATCCATGCAAAACTGA	57
miR-16-5p	TAGCAGCACGTAAATATTGGCG	57
miR-30d-5p	TGTAAACATCCCCGACTGGAAG	58
miR-216b-5p	AAATCTCTGCAGGCAAATGTGA	56
miR-765	TGGAGGAGAAGGAAGGTGATG	57
miR-105-5p	TCAAATGCTCAGACTCCTGTGGT	60
miR-608	AGGGGTGGTGTTGGGACAGCTCCGT	71
U6	CTCGCTTCGGCAGCACA	58
miR-3144-3p	ATATACCTGTTCGGTCTCTTTA	51
miR-302d-3p	TAAGTGCTTCCATGTTTGAGTGT	55
miR-26a-5p	TTCAAGTAATCCAGGATAGGCT	54
miR-26a-5p	TTCAAGTAATCCAGGATAGGCT	54
let-7a-5p	UGAGGUAGUAGGUUGUAUAGUU	51
Universal reverse	GCAGGGTCCGAGGTATTCG	60
